# The burden of health expenditure on household impoverishment in Ethiopia: a systematic review and meta-analysis

**DOI:** 10.1186/s12962-024-00543-2

**Published:** 2024-05-04

**Authors:** Yawkal Tsega, Abel Endawkie, Shimels Derso Kebede, Natnael Kebede, Mengistu Mera Mihiretu, Ermias Bekele, Kokeb Ayele, Lakew Asmare, Fekade Demeke Bayou, Mastewal Arefaynie

**Affiliations:** 1 Department of Health System and Management, School of Public Health, College of Medicine and Health Sciences, Wollo University, Dessie, Ethiopia; 2 Department of Epidemiology and Biostatistics, School of Public Health, College of Medicine and Health Sciences, Wollo University, Dessie, Ethiopia; 3 Department of Health Informatics, School of Public Health, College of Medicine and Health Sciences, Wollo University, Dessie, Ethiopia; 4 Department of Health Promotion, School of Public Health, College of Medicine and Health Sciences, Wollo University, Dessie, Ethiopia; 5 Department of Reproductive and Family Health, School of Public Health, College of Medicine and Health Sciences, Wollo University, Dessie, Ethiopia

**Keywords:** Impoverishment, Health expenditure, Ethiopia, Systematic review and meta-analysis

## Abstract

**Background:**

Ethiopia, like many low-income countries, faces significant challenges in providing accessible and affordable healthcare to its population. Health expenditure is a critical factor in determining the quality and accessibility of healthcare. However, high health expenditure can also have detrimental effects on households, potentially leading to impoverishment. To the best knowledge of investigators, no similar study has been conducted in Ethiopia. Therefore, this systematic review and meta-analysis aimed to determine the pooled burden of health expenditure on household impoverishment in Ethiopia.

**Methods:**

This systematic review and meta-analysis used the updated Preferred Reporting Items for Systematic Reviews and Meta-Analyses (PRISMA) guideline. PubMed, Cochrane Library, HINARI, Google Scholar and Epistemonikos electronic databases were searched systematically. Moreover, direct manual searching through google was conducted. The analysis was performed using STATA version 17 software. Heterogeneity and publication bias were assessed using I^2^ statistics and Egger’s test, respectively. The trim and fill method was also performed to adjust the pooled estimate. Forest plots were used to present the pooled incidence with a 95% confidence interval of meta-analysis using the random effect model.

**Results:**

This systematic review and meta-analysis included a total of 12 studies with a sample size of 66344 participants. The pooled incidence of impoverishment, among households, attributed to health expenditure in Ethiopia was 5.20% (95% CI: 4.30%, 6.20%). Moreover, there was significant heterogeneity between the studies (I^2^ = 98.25%, P = 0.000). As a result, a random effect model was employed.

**Conclusion:**

The pooled incidence of impoverishment of households attributed to their health expenditure in Ethiopia was higher than the incidence of impoverishment reported by the world health organization in 2023.

## Background

When medical expenses push households below a certain poverty level or push already impoverished households into extreme poverty, it is called impoverishing health expenditure (IHE) [1]. The World Health Organization (WHO) showed that, although financial risk protection (FRP) of households has been prioritized by healthcare systems of its member states, households' out-of-pocket (OOP) health expenses account for an unacceptably greater share of healthcare costs in low income countries like Ethiopia [2–12].

Globally, over 100 million individuals from 25 million households have been driven into poverty due to their healthcare expenses. Moreover, in 2019, 344 million people faced impoverishing health spending at the extreme poverty line ($2.15 a day extreme poverty line) [12]. Likewise, in 15 African nations, approximately one-third of the population had to borrow money and sell assets to cover their medical expenses. This financial strain increases to 50% when addressing the costs of hospitalization and 40% when seeking various other medical services [2, 5, 10, 12–14].

Moreover, when households face overwhelming medical costs, they are compelled to forego their basic necessities, such as food and education. If this situation persists over an extended period, households are inevitably driven toward poverty and deeper levels of absolute poverty [2, 12, 13, 15, 16].

Furthermore, household out-of-pocket healthcare spending is a barrier to households' access to essential healthcare services, but it is also one of the indicator used to assess and address the level of financial risk protection, one of the sustainable development goal(SDGs) and Ethiopian Health Sector Transformation Plan two(HSTP II) priorities, in healthcare systems [6–11, 15–17].

Ethiopia, like many low-income countries, faces significant challenges in providing accessible and affordable healthcare to its population. Health expenditure, which refers to the amount of money spent on healthcare goods and services, is a critical factor in determining the quality and accessibility of healthcare. However, high health expenditure can also have detrimental effects on households, potentially leading to impoverishment [18].

In Ethiopia, where a significant portion of the population lives below the poverty line, the impact of health expenditure on household impoverishment is a pressing concern. High out-of-pocket payments for healthcare can push households further into poverty, as they may be forced to sell assets, borrow money, or cut back on other essential expenses to cover medical costs. Understanding the relationship between health expenditure and household impoverishment is crucial for policymakers and healthcare providers to develop effective strategies to mitigate the negative consequences [1, 18].

Conducting a systematic review and meta-analysis is essential to synthesize existing evidence on the effect of health expenditure on household impoverishment in Ethiopia. By systematically reviewing and analyzing available studies, we can gain a comprehensive understanding of the relationship between these two variables, health expenditure and household impoverishment. Moreover, to the best of authors’ knowledge, no systematic review and meta-analysis have been carried out on the burden of health expenditure on household impoverishment in Ethiopia. Therefore, the objective of this study is to assess the burden of health expenditure on the impoverishment of households in Ethiopia. By addressing this research question, we can provide valuable insights into the impact of health expenditure on household well-being and inform policy decisions aimed at reducing poverty and improving healthcare access in Ethiopia. The research question of the review was: what was the incidence impoverishment attributed to health expenditure among households in Ethiopia?

## Methods

### Reporting and registration of the protocol

The report of this systematic review and meta-analysis (SRMA) was written using the latest Preferred Reporting Items for Systematic Review and Meta-Analysis 2020 (PRISMA 2020) guideline. The protocol of this SRMA has been recorded in the International Prospective Register of Systematic Reviews (PROSPERO) database with a reference number of CRD42023443529.

### Eligibility criteria

#### Inclusion criteria

*Study type* All observational studies (cross-sectional, and cohort) and gray literatures reporting the burden of health expenditure on households impoverishment in Ethiopia were included.

*Setting* Both institutional and community based studies conducted either on cost of specific or all health service in Ethiopia were included.

*Language* Articles written and published only in English language were included.

*Study year* All articles were incorporated into the study regardless of their publication and study year.

#### Exclusion criteria

We excluded articles with unreliable data, did not report the desired outcome, had different outcomes of interest, and were based on randomized controlled trials. Additionally, we excluded systematic reviews and meta-analyses, case reports, viewpoints, case series, books, articles without abstracts or with only abstracts as preceding papers, and conference papers. We also excluded articles conducted on nonhuman subjects, articles with qualitative designs, and studies published in languages other than English.

#### Information sources

This systematic review and meta-analysis extracted information from electronic databases including PubMed, HINARI, Cochrane library, Google scholar, semantic scholar, and Epistemonikos. Moreover, institutional repositories of Ethiopian Universities and google search engines were searched. The searches were conducted from October 1 to 30/2023.

#### Search strategies

A systematic literature search was conducted to identify relevant studies on the burden of health expenditure on household impoverishment in Ethiopia. Databases such as PubMed, Cochrane library, HINARI, Epistemonikos, Semantic Scholar, and Google Scholar were used. Moreover, Google and Ethiopian Universities institutional repositories were explored through direct manual search. The articles were searched using appropriate keywords and Boolean operators by combining as Impoverishment [Title/Abstract] OR Poverty [Title/Abstract] AND “Health Expenditure” [Title/Abstract] OR “Health Payment” [Title/Abstract] AND Ethiopia [Title/Abstract]. Finally, an additional search was conducted on the Google search engine and snowball searching manually. We exported the articles retrieved from different electronic databases and manual searches to EndNote version X7 reference manager software. We removed duplicates due to differences in reference styles among the sources using Endnote software and manually through assessing the full text. Throughout the process, we maintained a record of all the articles we reviewed.

#### Study records

##### Data management

We followed an updated PRISMA 2020 guideline to review the identified articles by their titles, abstracts, as well as full-text contents against the predefined eligibility criteria [19].

##### Selection process

Two independent reviewers (YT and NK) chose the articles through the following two stages of the selection process. Eligibility of the research was determined in the first step using titles and abstracts. In the second phase, the whole texts were evaluated in relation to the pre-set eligibility requirements. Articles that were confusing, ambiguous, incomplete, or devoid of any pertinent information were excluded throughout the article selection process. Moreover, the corresponding author of articles, no full text accessed freely, was communicated through email at least twice, in two weeks apart, in order to obtain the missing information prior to excluding the article.

#### Data collection process /data extraction/

Relevant data from each eligible study were extracted using customized and standardized Microsoft Excel 2013 form containing author, publication year, study year, study design, sample size, response rate, poverty line and the desired outcome variable. Both reviewers (YT and NK) evaluated the extracted data and determined the level of evidence. Any differences in opinion between the two independent reviewers were resolved by consensus-based discussion. If agreement was not reached, a third party (AE) was engaged to handle the disagreements.

#### Data items /definition of variables/

*Systematic review* was defined as a review of the available evidence on a formulated research question using an explicit plan and systematic search strategy.

*Meta-analysis* was defined as a statistical method used to pool or systematically combine, summarize, and interpret the results from more than one previous primary study to derive conclusions about that body of research.

*Household health expenditure* was defined as the sum of household expenses directly or indirectly to get any type of healthcare services from private, public or traditional institutions.

#### Outcome measurement

The pooled incidence of impoverishment due to health expenditure in Ethiopia was determined as the main outcome of this systematic review and meta-analysis. Impoverishment, due to health expenditure, is defined as if the households pushed below a given poverty line because of their health expenditure.

#### Risk of bias in individual studies /quality assessment/

In order to assess the quality of the articles, the trustworthiness and relevance of the results were evaluated using standardized critical appraisal tools from the Joanna Briggs Institute (JBI). Studies with a quality score of greater than 50% (four out of eight) for cross-sectional studies and 50% (six out of twelve) for cohort studies in JBI's standardized critical appraisal were considered low risk and included in this systematic review and meta-analysis.

#### Data synthesis and analysis

Selected articles were rated for level of evidence, methodological quality, and information that included. Based on the evidence compiled, answers to the targeted questions were formulated and anonymized voting was done. A level of consensus of 80% or higher was considered to represent a strong agreement among reviewers. Subsequently, selected studies data were qualitatively synthesized. Moreover, meta-analysis was done using Stata Version 17 software. A descriptive statistical analysis was performed to summarize the extracted data with their respective components. The standard error of incidence for each original article was calculated using binomial distribution formula. Forest plot was used to summarize information on each study and show the estimated common effect, and all in one figure. Furthermore, Cochrane’s Q statistics and I^2^ test were used to check whether heterogeneity across included articles statistically. Because of heterogeneity detected across included studies, a random effect model was employed. Subgroup analyses were carried out based on study year (recent/outdated), and scope of studies (general/specific). Sensitivity analysis was also conducted to assess the effects of a single study on incidence of impoverishment due to health expenditure.

##### Meta-bias/es

Meta-bias, presence of potential publication bias, and selective reporting within articles were assessed subjectively by using a Funnel plot through a visualization of the symmetry of the amount of study heterogeneity and egger test to check publication bias statistically.

## Results

### Search results

**Fig. 1:**
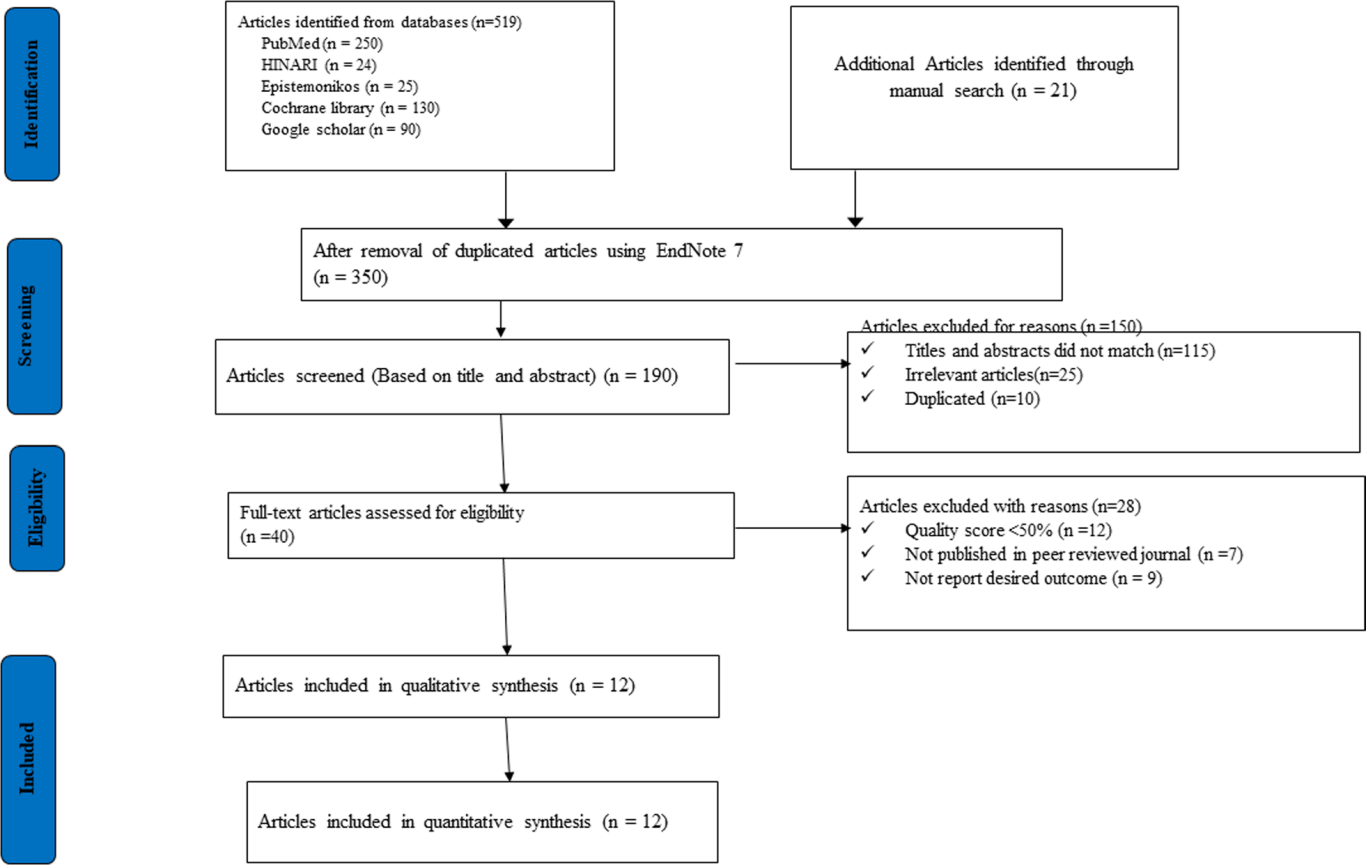
PRISMA study flow diagram describing the selection of articles for the systematic review and meta-analysis of the burden of health expenditure on household impoverishment in Ethiopia, 2023

A total of 540 studies were found, 519 from databases and 21 through manual searches. After removing duplicates using EndNote X7 reference manager software, 350 studies were excluded. The remaining 190 articles were assessed based on their titles and abstracts, with 150 studies being excluded for various reasons. Then, 40 full text studies were critically appraised, leading to the exclusion of 28 additional studies that did not meet the predetermined inclusion and exclusion criteria. Finally, 12 articles with a total of 66,344 participants were included in the systematic review and meta-analysis to determine the burden of health expenditure on household impoverishment in Ethiopia. Eventually, 12 articles were eligible for meta-analysis (Table 1, Fig. 1).

**Table 1:** Descriptive summary of 12 studies reporting the burden of health expenditure on household impoverishment in Ethiopia, 2023

First author(year)(refs.)	Study area	Study design	Sample size	Quality score	Risk
Alemayehu et al. [20]	National	Cross-sectional	4238	7	Low
Borde et al. [30]	SNNP	Cohort study	896	8	Low
Getachew et al. [21]	Oromia	Cross-sectional	633	7	Low
Hailemichael et al. [22]	SNNP	Cross-sectional	257	8	Low
Kiros et al. [23]	National	Cross-sectional	30229	8	Low
Tsega et al. [1]	Amhara	Cross-sectional	423	7	Low
Obse and Ataguba [24]	National	Cross-sectional	28032	7	Low
Tsega et al. [25]	Amhara	Cross-sectional	422	7	Low
Tamirat [26]	SNNP	Cross-sectional	403	6	Low
Shigut [27]	Oromia	Cross-sectional	334	5	Low
Kindeneh [29]	Amhara	Cross-sectional	422	6	Low
Assebe LF et al. [28]	Oromia	Cross-sectional	221	7	Low

### Characteristics of included articles

Eleven studies utilized cross-sectional study design [1, 20–29]. Whereas, one study used longitudinal cohort study design [30]. Likewise, the with regard to the location of studies, three studies were conducted at national level [20, 23, 24], three in Amhara region [1, 25, 29], three in Oromia region [21, 27, 28], and three studies were conducted in SNNP region [22, 26, 30]. Moreover, 6 studies estimated the cost of all diseases (i.e. studies conducted either using nationally representative survey data or community level collected data regarding all healthcare service costs) [1, 20, 21, 23, 24, 27]. However, 6 studies took into account the cost of specific healthcare service [22, 25, 26, 28–30]. Furthermore, the studies were conducted from 2011 to 2022 and published from 2018 to 2023 (Table 1).

### Risk of bias within articles

By using Joanna Briggs Critical Appraisal Tools for review and meta-analysis for cross-sectional articles and cohort articles, those studies that had low risk were included for the review.

### The burden of health expenditure on household impoverishment

The overall analysis of 12 studies revealed that the mean burden of health expenditure on household impoverishment was 10.60% (95%CI 0.30, 20.88%) with standard deviation of 16.19% in Ethiopia.

### Pooled incidence of impoverishment due to health expenditure

**Fig. 2:**
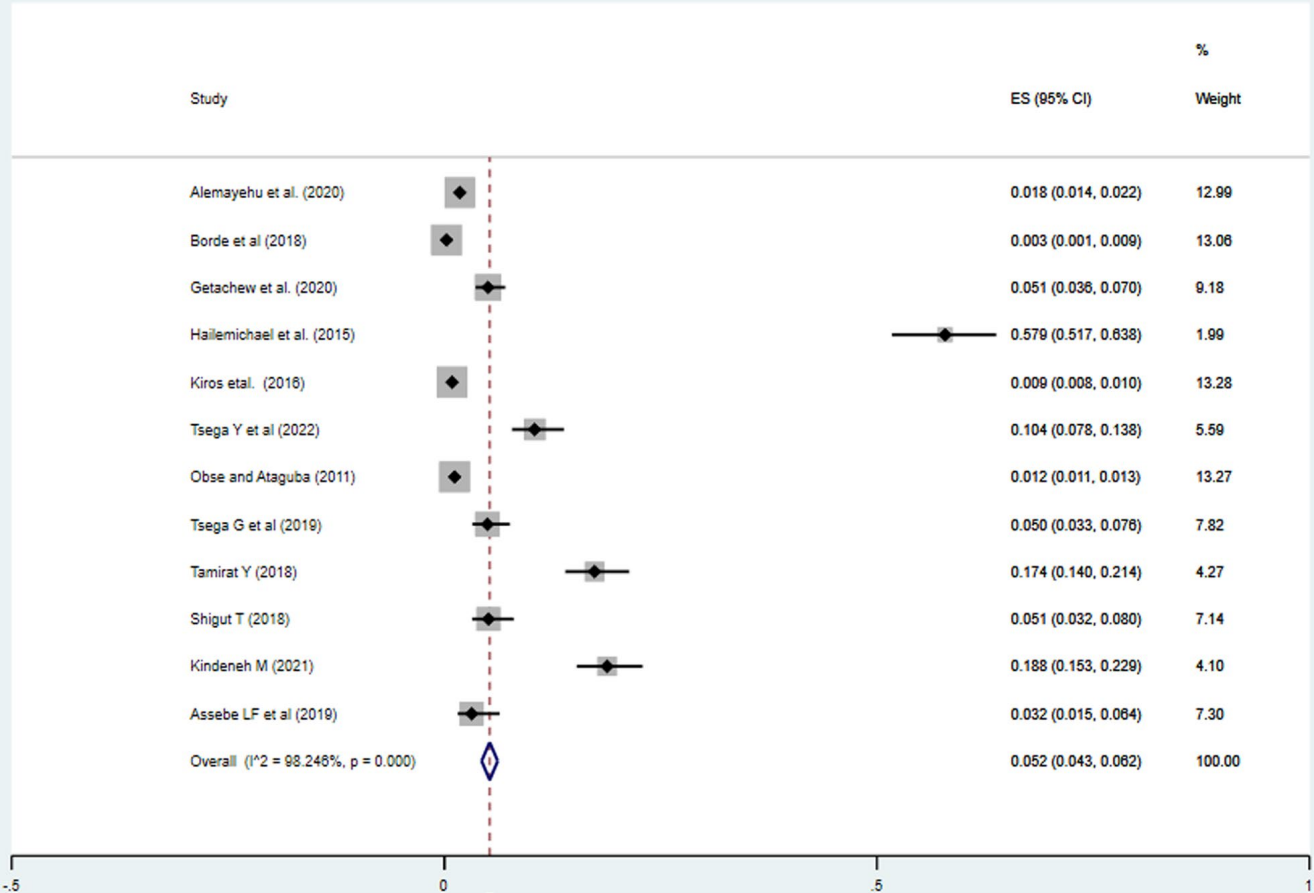
Meta-analysis of the pooled incidence of impoverishment of households due to health expenditures in Ethiopia, 2023

The pooled incidence of impoverishment attributed to health expenditure among households in Ethiopia was 5.2% (95%CI: 4.3%, 6.2%). Moreover, there was a significant heterogeneity between the studies (I^2^ = 98.25%, P = 0.000). As a result, a random effect model was employed to estimate the pooled incidence of impoverishment due to healthcare expenditures in Ethiopia (Fig. 2).

### Subgroup analysis

Since there is a significant heterogeneity across studies, subgroup analysis has been employed in this meta-analysis based on scope (general/specific), response rate (high/low), and risk of bias (high/low). However, there was still significant heterogeneity among studies within the subgroups.

#### Scope of the studies

**Fig. 3:**
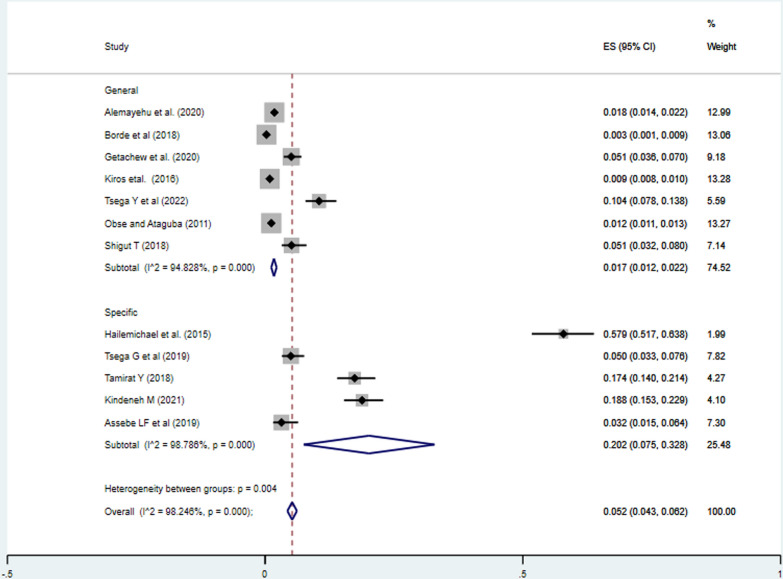
Meta-analysis on incidence of impoverishment attributed to health expenditure for the scope of the studies subgroup analysis in Ethiopia, 2023

The incidence of impoverishment in Ethiopia with studies estimating costs of all healthcare services (general cost) was 1.70%( 95%CI: 1.20%, 2.20%), lower than the studies estimating the cost of specific healthcare cost (specific cost), 20.20% (95%CI: 7.60%, 32.80%) (Fig. 3).

#### Response rate of the studies

**Fig. 4:**
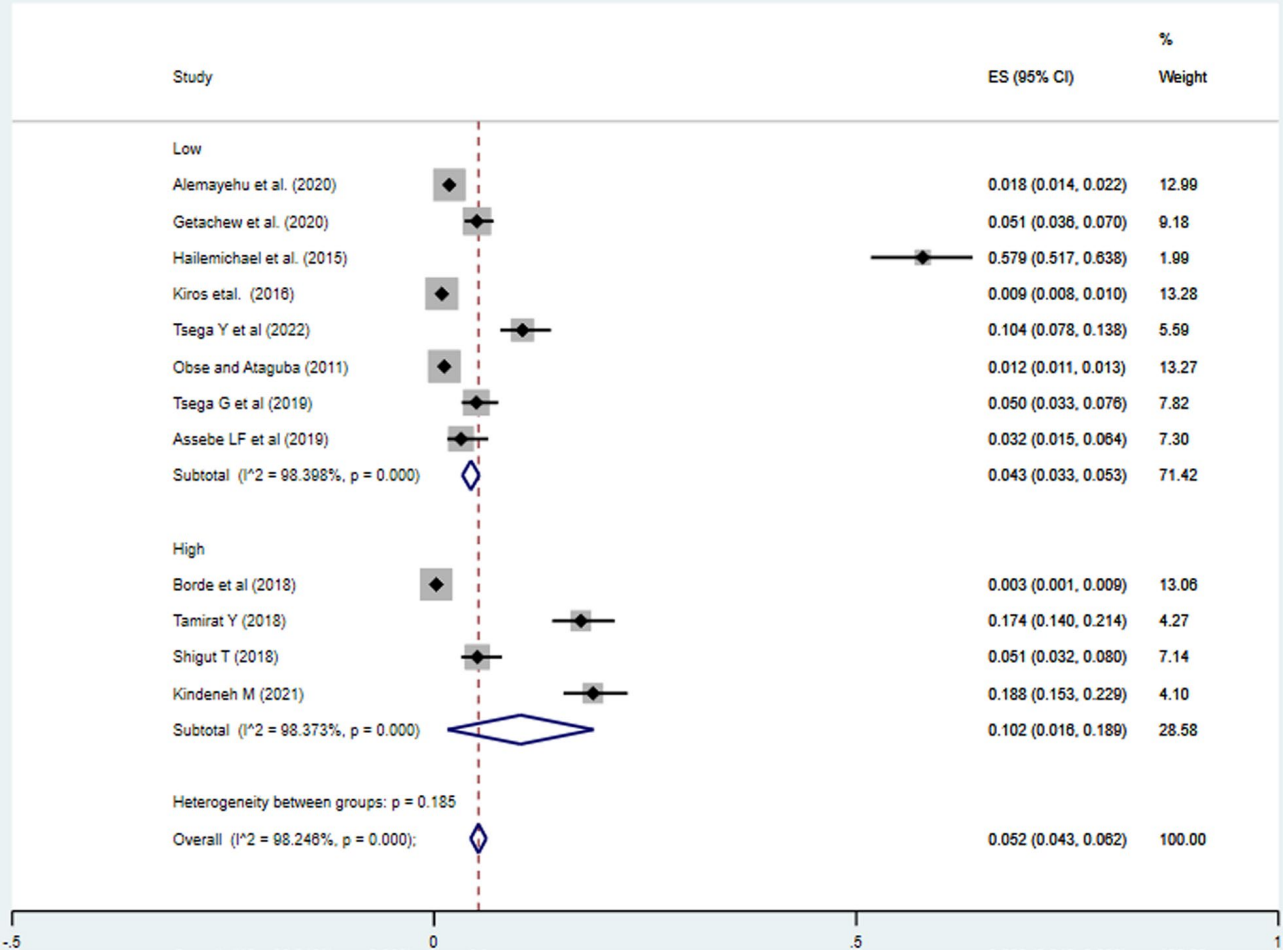
Meta-analysis on incidence of impoverishment attributed to health expenditure the response rate subgroup analysis in Ethiopia, 2023

The incidence of impoverishment in studies with high response rate was 2.40% (95%CI: 1.80%, 3.00%), lower than the incidence of impoverishment due to health expenditure in studies with low response rate, 18.00% (95%CI: 7.10, 29.00%) (Fig. 4).

#### Risk of bias of the studies

**Fig. 5:**
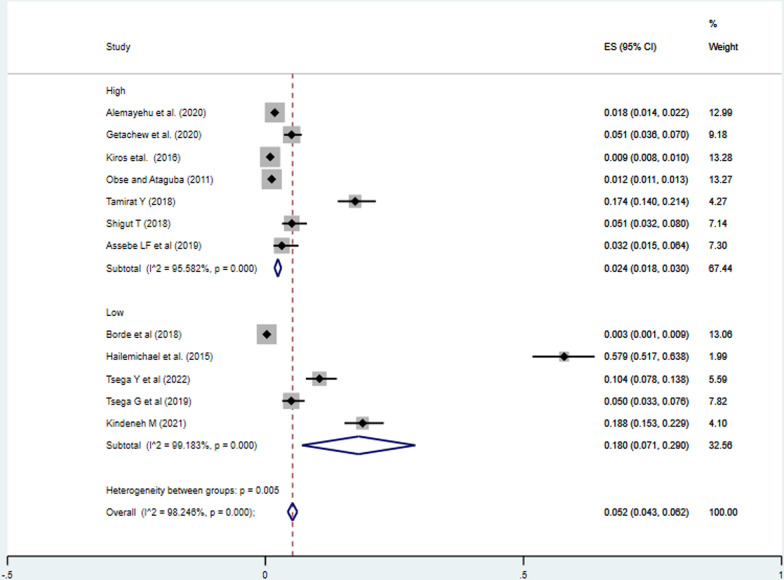
Meta-analysis on incidence of impoverishment attributed to health expenditure for the risk of bias of studies subgroup analysis in Ethiopia, 2023

The incidence of impoverishment attributed to health expenditure among studies with low risk of bias was 4.30% (95%CI: 3.30%, 5.30%), lower than the incidence of impoverishment among studies with high risk of bias which was 10.20%( 95%CI: 1.63, 18.00%) (Fig. 5).

#### Publication bias

**Fig. 6:**
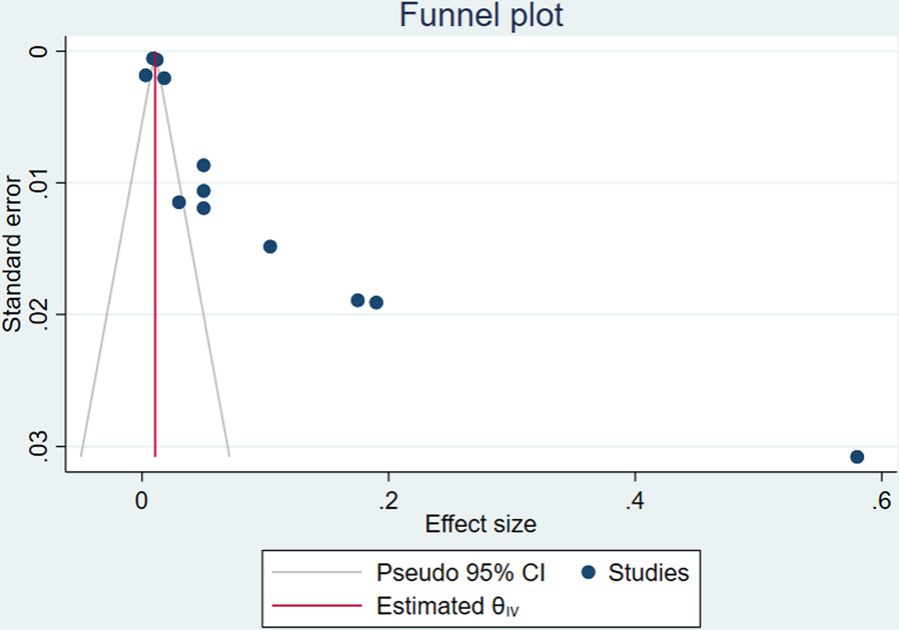
Publication bias across included studies in Ethiopia, 2023

There was statistically significant publication bias across included studies (p <0.001). The funnel plot of publication bias was shown in Fig. 6.

## Discussion

This systematic review and meta-analysis (SRMA) aimed to determine the pooled incidence of impoverishment attributed to health expenditure in Ethiopia. The pooled incidence of impoverishment caused by health expenditure among households in Ethiopia was 5.20% (95% CI: 4.30%, 6.20%). Furthermore, this SRMA revealed that the incidence of impoverishment due to health expenditure among studies estimating only the cost of specific disease or health service was higher than the incidence of impoverishment among studies estimating the cost of whole medical health services in Ethiopia.

Likewise, the findings of this systematic review and meta-analysis implied that health care expenditure is still a major public health problem, which has a significant role in increasing poverty in Ethiopia. Moreover, such impoverishing health expenditure obliged households to leave other subsistence needs (like school, food), sell their important assets (like home, cattle and jewelries) and forgone essential health services, which increases mortality and morbidity within the community and make countries less productive in their short and long time oriented activities. Therefore, policymakers in Ethiopia need to address the issue of high health expenditure and its impact on household impoverishment, should consider implementing strategies such as community-based health insurance, social health insurance, exempted services, and fee waiver systems to safeguard households from financial catastrophe due to healthcare spending.

The pooled incidence of impoverishment due to health spending (i.e. 5.2%) in this SRMA was lower than the 2021(i.e. 6.7%) and higher than the recent 2023 World Health Organization reports (i.e. 4.4%) [12, 15, 16] in tracking and monitoring universal health coverage in WHO member states. The possible reason behind the discrepancy might be due to the fact that the difference in the scope of the study setting and WHO report incorporate developed nations, which have better financial risk protection platforms. The other probable reason might be due to the current study incorporating the recent estimated health care costs and the time itself may have a great significant impact, difference in the cost of healthcare costs.

## Limitation of the study

The potential limitations of this study both at the study and systematic review level are the different poverty lines used to estimate incidence of impoverishment due to health expenditure, and the presence of significant heterogeneity across included studies although subgroup analysis has been employed, there is still a substantial heterogeneity. Moreover, publication bias was also revealed in this systematic review and meta-analysis.

## Conclusions

The pooled incidence of impoverishment in Ethiopia was higher than the incidence of IHE reported by the World health organization in 2023. In addition, the incidence of impoverishment was higher in the studies that estimated the cost of a single illness or medical treatment than the incidence of impoverishment in the studies estimating the cost of all medical expenditures in Ethiopia. Therefor, the concerned body, Ethiopian Federal Ministry of Health, better to be committed to protect the  households from financial risk of health expenditure. 

## Data Availability

All relevant data are found in the manuscript.

## Abbreviations

IHE: Impoverishing health expenditure
PRISMA: Preferred reporting items for systematic review and meta-analysis
OOP: Out of pocket
SDG: Sustainable development goals
FRP: Financial risk protection
WHO: World Health Organization
